# Functionalised-biomatrix for wound healing and cutaneous regeneration: future impactful medical products in clinical translation and precision medicine

**DOI:** 10.3389/fbioe.2023.1160577

**Published:** 2023-05-24

**Authors:** Nur Izzah Md Fadilah, Shaima Maliha Riha, Zawani Mazlan, Adzim Poh Yuen Wen, Looi Qi Hao, Blessy Joseph, Manira Maarof, Sabu Thomas, Antonella Motta, Mh Busra Fauzi

**Affiliations:** ^1^ Centre for Tissue Engineering and Regenerative Medicine, Faculty of Medicine, Universiti Kebangsaan Malaysia, Kuala Lumpur, Malaysia; ^2^ Department of Surgery, Hospital Canselor Tuanku Muhriz, Universiti Kebangsaan Malaysia, Kuala Lumpur, Malaysia; ^3^ My Cytohealth Sdn Bhd Kuala Lumpur, Kuala Lumpur, Malaysia; ^4^ Business Innovation and Incubation Centre, Mahatma Gandhi University, Kottayam, Kerala, India; ^5^ International and Inter University Centre for Nanosciences and Nanotechnology, Mahatma Gandhi University, Kottayam, Kerala, India; ^6^ Department of Industrial Engineering, University of Trento, Trento, Italy

**Keywords:** multifunctional bioscaffolds, skin substitutes, biomaterials, wound healing, skin tissue engineering

## Abstract

Skin tissue engineering possesses great promise in providing successful wound injury and tissue loss treatments that current methods cannot treat or achieve a satisfactory clinical outcome. A major field direction is exploring bioscaffolds with multifunctional properties to enhance biological performance and expedite complex skin tissue regeneration. Multifunctional bioscaffolds are three-dimensional (3D) constructs manufactured from natural and synthetic biomaterials using cutting-edge tissue fabrication techniques incorporated with cells, growth factors, secretomes, antibacterial compounds, and bioactive molecules. It offers a physical, chemical, and biological environment with a biomimetic framework to direct cells toward higher-order tissue regeneration during wound healing. Multifunctional bioscaffolds are a promising possibility for skin regeneration because of the variety of structures they provide and the capacity to customise the chemistry of their surfaces, which allows for the regulated distribution of bioactive chemicals or cells. Meanwhile, the current gap is through advanced fabrication techniques such as computational designing, electrospinning, and 3D bioprinting to fabricate multifunctional scaffolds with long-term safety. This review stipulates the wound healing processes used by commercially available engineered skin replacements (ESS), highlighting the demand for a multifunctional, and next-generation ESS replacement as the goals and significance study in tissue engineering and regenerative medicine (TERM). This work also scrutinise the use of multifunctional bioscaffolds in wound healing applications, demonstrating successful biological performance in the *in vitro* and *in vivo* animal models. Further, we also provided a comprehensive review in requiring new viewpoints and technological innovations for the clinical application of multifunctional bioscaffolds for wound healing that have been found in the literature in the last 5 years.

## 1 Introduction

Skin, the “first line of defense” in the human body, acts as a shield against the external environment and assists in thermal regulation and hydration retention (Bacakova et al., 2016). In addition, it helps to prevent microbial attack via infiltration of immune cells, including neutrophils or macrophages, and restoring damaged tissue function through rapid regeneration (Chaudhari et al., 2016). However, any deep partial-thickness or full-thickness skin wounds >4 cm like diabetic ulcer and burn usually take a longer time to heal. This is due to a lack of epithelialisation foci from hair follicles, sweat glands, and other dermal appendages and requires additional surgery, necessitating the utmost requirements of skin substitute for tissue repair and regeneration (Bhardwaj et al., 2018). Any damage to this tissue represents a substantial imbalance of physiological processes that may lead to mortality, hospitalisation, or long-time morbidity (Korrapati et al., 2016). In response to the injury, most skin wounds like skin cuts heal naturally by stopping hemorrhage, and avoiding excessive blood loss leading to death. Additionally, skin and subcutaneous disorders were rated as the fourth most common cause of non-fatal disease burden globally, highlighting the importance of dermatology in the rapidly developing field of global health (Hay et al., 2014). According to Rachel et al. (Giesey et al., 2021), skin and subcutaneous disease grew from 46.8% between 1990 and 2017 and is ranked fourth by the incidence of all causes of disease. There is global variation in disease burden when stratified by age, sex, geographic regions, and sociodemographic index.

In the past, the split skin graft (SSG) method was used to correct skin defects by harvesting skin from parts of the body that had not been harmed. This skin had the entire epidermal layer as well as a small amount of the dermal layer. The role of SSG is to direct self-renewing keratinocyte stem cell proliferation to the injured location for skin regeneration. However, due to a lack of donor sites and keloid formation, the use of SSG in full-thickness skin wound treatment is currently restricted. In addition, collecting skin grafts from severe burns will result in fresh wounds and more physiological harm (Shimizu and Kishi, 2012). In order to cure skin defects such as diabetic ulcers and burn injuries, engineered skin substitute (ESS) developed as a promising therapeutic option to conventional dressings and autologous skin grafts (Kennedy et al., 2017). Despite showing promising results in wound healing applications, ESS bears the threat of causing further infection at the wound site that could elevate the severity of the patient’s health condition.

**FIGURE 1:**
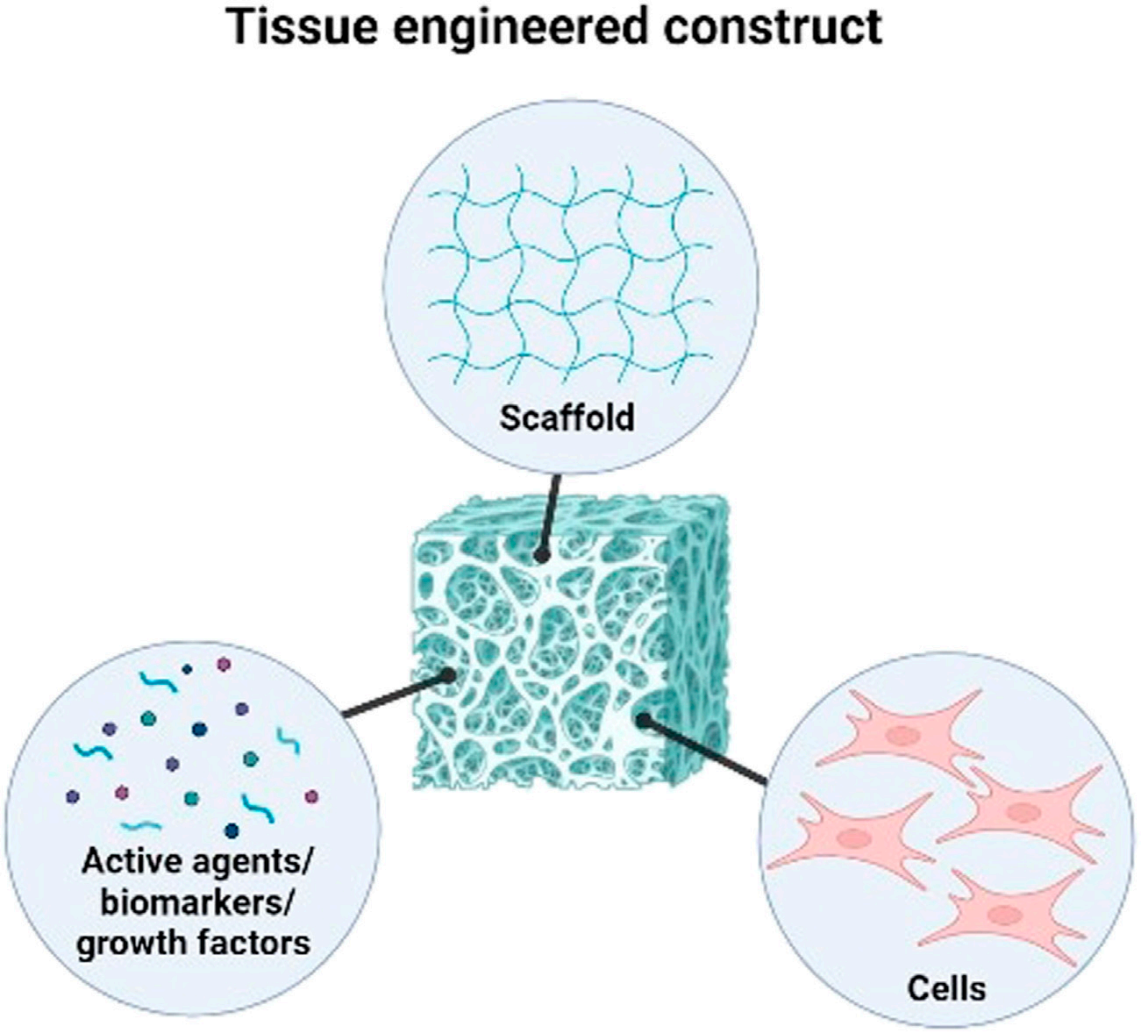
Three key elements in combining to form tissue engineered construct for tissue engineering and regenerative medicine.

The main component of conventional tissue engineering approaches is defined as the use of a scaffold as a structural element with well-defined physical, chemical, mechanical, and biological properties, as well as the right structure and porosity to support the metabolism and healing mechanisms specific to the cell tissue (Kaliva et al., 2017). Decellularised allogeneic dermis, reconstituted collagen gels and sponge, natural and synthetic polymer films make up the components of commercially available skin substitutes. However, these foreign bodies necessitate several transplantation procedures. The lack of the structural and biological cues necessary to promote fast vascularisation and regenerative cell propensity after implantation into the patient’s body may cause prolonged inflammation leading to immunological rejection. In tissue engineering approaches, the construction of a multifunctional scaffold is focused on combining three key elements to address these limitations; biomaterials as a microenvironment to trigger and guide specific cells bioactivity necessary for tissue development eventually loaded with an appropriate cells type, active agents, nanoparticles (NPs) and/or biomolecules (Alaribe et al., 2016; Fadilah et al., 2022a) (Figure 1).

The term multifunctional bioscaffolds refers to a three-dimensional (3D) structure that consists of various combinations of bioactive agents, bioinert components, and molecules to improve cells biomaterial contacts, prevent infections while biodegrading at a certain controlled-rate, overall contributing to skin regeneration (Swartjes, 2017). The properties and behavior of multifunctional bioscaffolds made from various natural and synthetic biomaterials can be tuned, and they can carry out multiple tasks at once, including delivering bioactive agents and pharmaceutical molecules, controlling stem cells behavior, and directing cells growth and differentiation (Qazi et al., 2015; Fadilah et al., 2021). The development of multifunctional bioscaffolds that can actively engage in the process of providing the biological signals that guide and drive cells activity (attachment, proliferation, migration, growth, and differentiation) is thoroughly researched using organic, inorganic, and hybrid (organic-inorganic) materials (Gupta et al., 2022; Selvakumar and Lonchin, 2022). Using multifunctional bioscaffolds, various degrees of success in skin regeneration has been demonstrated *in vitro* and *in vivo* models (Li et al., 2018a; Rahmanian et al., 2019; Ramadass et al., 2019; Abdel-Mohsen et al., 2020; Yang et al., 2020a; Sallehuddin et al., 2022). Though, just a few of these approaches outlined above have been applied to human clinical trials to be commercialised. This is because the fabrication of multifunctional bioscaffolds for clinical use requires controllable properties with uniform porous 3D structure, interconnected porosity, and proper mechanical properties to carry cells and bioactive molecules for wound healing and skin regeneration (Zhao et al., 2017). Additionally, to our knowledge several factors might contribute to the failure of clinical trials instead of the effect of implanted multifunctional bioscaffolds. Proper planning for execution is essential to ensure the integrity and related procedures can take place smoothly. To accomplish the whole activity, the sustainability of machinery and facilities for a planned clinical trial should be in place throughout the particular time frame. Any critical trial should dedicate a competent and permanent data collector to securely keep all the data information before further analysis. Besides, the difficulty in patient recruitment is because of the stringent inclusion criteria which could limit the success rate of any clinical trial. The major adverse event of the implanted prodder such as safety issue, may contribute the most to clinical trial failure. However, the successful implantation into the targeted area needs to strategies for effective post-implantation arrangement mainly for trial participants (Fadilah et al., 2022b). It includes knowledge about motivation, good dressing, and proper education that emphasises post-application care or post-operative care (Gizaw et al., 2022). For example, proper dressing and splinting are essential for skin wound care management to immobilize the specific area post-operative. The splinting can assist in managing better wound healing, including vacuum or back slab splint.

The range of biomaterials, either in natural or synthetic polymers and their composites incorporated with bioactive agents like growth factors have been considered in several reviews to promote skin regeneration (Follmann et al., 2017; Sharma et al., 2019; Mertgen et al., 2020). This review emphasises how cells including fibroblasts and stem cells, interact with traditional bioscaffolds to encourage their growth, migration, and differentiation toward skin regeneration. The geometric structure, mechanical, and biochemical characteristics of the matrix, in addition to the biomaterials and biomolecules utilised, also control how cells behave. However, in recent years, special interest has been developed in multifunctional bioscaffolds with multiple wound healing properties and to ensure their translational potential for human clinical application. This is because wound healing requirements differ for each clinical application, making multifunctionality a prerequisite in most applications. For instance, ESS used in burn treatment not only needs to integrate with the native tissue without causing the problem, but also needs to prevent infection and promote improved recovery. Hence, there is a need for literature focusing on the impact of multifunctional bioscaffolds on wound healing. Therefore, in this review, we evaluated the commercial skin substitute products and their limitations, identifying key factors to improve the translation of multifunctional bioscaffolds for clinical settings as summarised in the graphical abstract in the Supplementary Materials. In addition, we evaluate the therapeutic results of these *in vitro* and pre-clinical multifunctional bioscaffolds research, and we discuss potential future directions for the advancement of multifunctional bioscaffolds towards clinical applications.

## 2 Data extraction management

A literature search was conducted within 5 years of publications (2018–2022) through the platform of PubMed, EBSCO host, Web of Science (WoS), Scopus, and Google Scholar. The search strategy used the terms of: “bioscaffolds,” “skin substitutes,” “biomaterials,” “wound healing,” “skin tissue engineering.” The exclusion criteria for this review would be all secondary literature and any original articles that have been wrote and submitted in other languages other than English.

## 3 Skin substitutes for treating wounds

Several factors, including the type of wounds (epidermal, deep dermal, full-thickness), the origin of tissue damage (first-, second-, or third-degree burn) or trauma, the amount of moisture in the wound, inflammation, and secondary infection, all play a role in the coordinated process of wound healing followed by skin regeneration (Fadilah et al., 2022). Skin wound healing is comprised of four phases (hemostasis, inflammation, proliferation, and wound remodeling), including the regeneration of the new cells induced via extracellular matrix (ECM) secretion by fibroblasts, followed by keratinocytes replication as well as layered-proliferation and finally, the differentiation of keratinocytes to form the outermost epidermis layer (Fadilah et al., 2020; Tottoli et al., 2020). For minor skin injuries to heal, simple cell contraction and proliferation within the wound site are required. On the other hand, more extensive skin wounds take much longer to heal and are more likely to experience unanticipated dangers, including inflammation, infection, and scarring, which can lead to chronic wounds (Negut et al., 2020). Furthermore, additional elements like illness states (such as diabetes and kidney infections), the presence of foreign bodies, malnutrition, an immunocompromised body, and older age may affect the process of tissue regeneration and wound healing (Guo and Dipietro, 2010). Hence, it is essential to consider these multifactorials (wound type and stages of wound healing) while developing engineered grafts for skin tissue regeneration.

**FIGURE 2:**
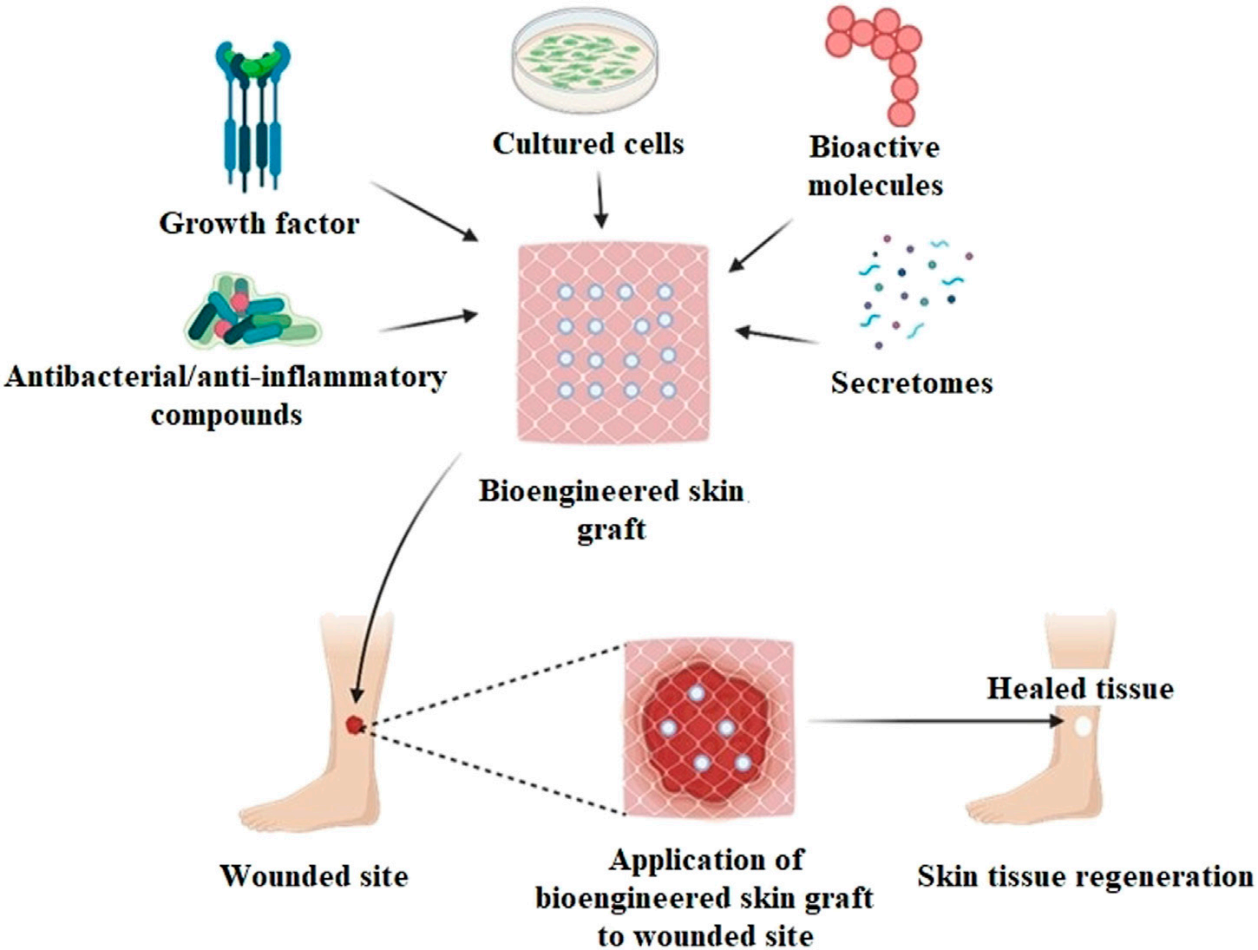
Bioengineered skin substitute (graft) for wound healing application.

Wound healing and skin tissue restoration have shown promising results in the last few years with the manufacture of novel skin tissue-engineered products. Various grafts (allo-, auto-, xeno-) of dermal, epidermal, or dermo-epidermal origin, such as Alloderm, Epicel, and OrCel have been used commercially and reported to exhibit improved wound healing and tissue regeneration efficiency (Chaudhari et al., 2016). Such grafts contributed to the regeneration of skin tissue structure by repairing the wound effectively. ESS is currently designed to repair wounds and provide supplements like bioactive molecules, growth factors, secretomes, antibiotics, and anti-inflammatory drugs, which eventually accelerate the process of wound healing (Figure 2). Multifunctional bioscaffolds have been developed as a part of these bioengineered substitutes to promote cells growth in 3D structures; which exhibit high biocompatibility and biodegradation, acting as a suitable graft for wounded skin tissue (Negut et al., 2020).

### 3.1 *In vitro* effect on cell sub-organelles

Biomatrices are frequently utilised in tissue engineering and regenerative medicine applications as to provide a scaffold for cells to adhere to and grow on it (Dhandayuthapani et al., 2011). The *in vitro* effect of a functionalized biomatrix on cell sub-organelles would differ depending on the specific type of functionalisation and the particular sub-organelles being studied. The functionalisation of the biomatrices can be done through various ways and methods, such as the incorporation of specific proteins, peptides, or growth factors, or the modification of surface chemistry to encourage cell adhesion and provide specific signaling cues (Krishna and Kiick, 2010; Tallawi et al., 2015; Mitchell et al., 2016).

Researchers may be interested in evaluating the changes in organelle morphology, localisation, or function with regard to the impact of a functionalised biomatrix on cell sub-organelles. One of the main organelles involved in the wound healing is the mitochondrion, which plays a critical role in energy production and cells metabolism (Yan et al., 2021). Another important organelle in the wound healing is the endoplasmic reticulum (ER), which is responsible for protein synthesis, folding, and transport. Changes in ER morphology and function have been observed in response to biomatrix functionalisation. For example, functionalisation of a biomatrix with a protein can have a significant effect on wound healing. One example is the use of ECM proteins such as collagen and fibronectin. It was known to promote mitochondrial biogenesis may lead to an increase in mitochondrial mass and improved mitochondrial function in cultured cells. Alternatively, modification of the ECM to mimic the basement membrane may promote the formation of tight junctions and increase the stability of the Golgi apparatus in the cultured cells (Jain et al., 2022). Fauzi et al. have found that collagen scaffolds can promote cell adhesion, migration, and proliferation, which are essential for the formation of new tissue (Fauzi et al., 2016; Busra et al., 2017; Mh Busra et al., 2019). Additionally, collagen can activate various signaling pathways that regulate the expression of genes involved in cell proliferation, ECM remodeling, and angiogenesis (Mathew-Steiner et al., 2021).

On the other hand, fibronectin is another ECM protein that is often used for biomatrix functionalisation in wound healing applications. It has been shown to promote cell adhesion and migration, as well as the formation of new blood vessels. It promotes the spreading of platelets at the site of injury, the adhesion and migration of neutrophils, monocytes, fibroblasts, and endothelial cells into the wound region, and the migration of epidermal cells through the granulation tissue. During matrix synthesis, fibronectin appears to be involved both in the organization of the granulation tissue and basement membrane. In terms of tissue remodeling, fibronectin functions as a nonimmune opsonin for phagocytosis of debris by fibroblasts, keratinocytes, and under some circumstances, macrophages (Grinnell, 1984).

In summary, the functionalisation of a biomatrix can have a significant impact on the morphology, localisation, and function of organelles involved in wound healing, such as mitochondria, ER, and Golgi apparatus. These changes can promote cellular energy production, ECM remodeling, and growth factor secretion, ultimately enhancing the process of skin tissue repair. However, *in vitro* studies can provide important insights into these effects, but further investigation will be needed to determine how they translate to the *in vivo* systems.

## 4 Commercially available skin substitutes

Whether the injury is acute or chronic, the optimal wound therapy in a clinical setting relies on the depth of the wound. Several commercial wound dressings can guard the wound site while maintaining the proper moisture levels and avoiding bacterial infection since they are based on polymer films, hydrogel, foams, sponges, and beads (Alven et al., 2022). However, the ECM modeling matrix and cells proliferation are not sufficiently provided by the wound dressings (Mele, 2016). In such cases, commercially available ESS is required (Table 1) to ensure proper epidermal, dermal, or/and full-thickness wound healing through inflammation, proliferation, and remodeling stage.

**TABLE 1:** Commercially available skin substitutes for wound healing applications.

Product name	Patent no.	Company	Component	Advantages	Disadvantages	Indications	References
	Dermal						
Alloderm®	EP1087756A1	Lifecell, Branchburg, NJ	Decellularised human dermis	Single-step procedure, no immunogenic reaction	Safety and ethical concerns for moral reasons	Burn injuries, soft tissue replacement	Bello et al. (2001); Gordley et al. (2009); Nathoo et al. (2014)
Integra®	D901737	Integra LifeScience, Plainsboro, NJ	Collagen–glycosaminoglycan matrix on a silicone membrane	Simple handling, a long shelf life, and low risks of immunogenic response	Double step-procedure, infection	Burn injuries, chronic injuries, soft tissue damage	Shevchenko et al. (2010); Gonzalez et al. (2020)
Biobrane®	EP2916876B1	Mylan Bertek Pharmaceuticals Inc., Canonsburg, PA	Porcine collagen-coated nylon mesh	Single step procedure	Insensitive to the contaminated wound bed	Partial- thickness burns	Cheah et al. (2014); Tan et al. (2015); Tavakoli and Klar (2021)
Terudermis	US20080095748A1	Terumo Co. Ltd., Japan	Lyophilised collagen sponge	Single-step grafting procedure; accelerated wound angiogenesis	7–14 days for the vessels to form into sponge	Burns and other traumatic and mucosal defects	Groeber et al. (2011); Hsu et al. (2021)
Dermagraft®	WO1999043787A2	Canton, MA	Polyglactin mesh seeded with allogeneic fibroblasts	Easy to handle, no rejection, and good tearing resistance	Infections, cellulitis, high cost, poor ECM structure	Diabetic foot and venous ulcers, burn and chronic injuries	Varkey et al. (2015)
	Epidermal						
Epicell®	US10004830B2	Genzyme Biosurgery, USA	Autologous keratinocytes attached to petrolatum gauze	A skin biopsy covers a large area	Unstable without a dermal replacement, costly	Deep dermal or full- thickness burns	Carsin et al., 2000; Supp (2011)
CellSpray	Not stated	Clinical Cell Culture (C3), Australia	Cultured/Non-cultured keratinocytes (subconfluent cell suspension)	Decreased cell culture time with earlier wound coverage	Limited to partial- thickness and graft donor site wounds	Chronic ulcers, burns	Magnusson et al. (2007); Goedkoop et al. (2010)
Lyphoderm	US7264826B2	XCELLentis NV, Belgium	Lyophilised neonatal keratinocytes	Prolonged shelf life and immediate availability	Multiple donors, difficult cell culture, and subsequent grafting	Venous leg ulcers	Harding et al. (2005)
Bioseed-S	Not stated	BioTissue Technologies GmbH, Germany	Keratinocytes in culture (subconfluent cell suspension)	Small and large surfaces	Patient biopsy is required, and cell expansion takes a long time	Burns	Bock et al. (2003)
MySkin	D753308	CellTran Ltd., UK	Keratinocytes in culture (subconfluent cell suspension)	Easier handling and application, reduced cell culture time, small and large surfaces	Patient biopsy required, several weeks delay, frail, and dermal support needed	Pressure and diabetic foot ulcers, superficial burns	Haddow et al. (2003)
	Dermal/epidermal composites						
Apligraf®	EP1984025A2	Organogenesis Inc., USA	Neonatal foreskin fibroblasts and keratinocytes seeded in a bovine collagen matrix	Enhanced healing after 4 weeks	Expensive, with a short shelf life and cautious handling	Venous and diabetic foot ulcers	Curran and Plosker (2002); Dodson and Levine (2015)
OrCel®	US-6500464-B2	Ortec International Inc., USA	Neonatal foreskin fibroblasts and keratinocytes seeded in a type I collagen matrix	Scarring and healing time is reduced	Plays a transitory role	Chronic wounds	Still et al. (2003); Stark et al. (2006)
Tiscover (A-Skin)™	Not stated	Advanced Tissue Medicinal Product, Netherlands	Full-thickness autologous cultured skin	No immune rejection	‘Of the shelf’ availability maybe limited due to the use of autologous cells	Chronic wounds, wounds resistant to therapy	Varkey et al. (2015)
DenovoSkin™	3174563	EUROSKINGRAFT, Univ. of Zurich, Switzerland	Full-thickness autologous replacement with dermal and epidermal layers	Near-normal skin structure	Long culture time, no recorded clinical series	Leg/foot ulcers resistant to chronic therapy	Varkey et al. (2015); Urciuolo et al. (2019)
Oasis®	K061711	Healthpoint Biotherapeutics, USA	Matrix intact from the submucosa of the porcine small intestine	Long shelf life and immediate availability	Little clinical data	Acute, chronic, and burn wounds	Hart et al., 2002; Límová (2010)

Despite improving wound healing, commercially available skin graft shows several limitations, such as low blood vessel formation, reduced mechanical strength, poor integration, scarring, and immune rejection (Alrubaiy and Al-Rubaiy, 2009). For instance, autologous grafts are considered a gold standard for full-thickness wounds; nevertheless, their use is restricted by the patient’s availability of healthy skin and necessitates additional surgery to remove donor tissue. Moreover, operative assessment on a ten-year-old boy using an autologous skin graft reported the development of progressive chest scarring, contraction and keloid formation with chronic pain limiting range of motion for the patient (Patterson et al., 2019). On the other hand, allogeneic grafts can act as a replacement, but the patient may suffer from immunological rejection and the risk of infection in the long run. The OrCel® (New York, NY) ESS is constructed by seeding allogeneic fibroblasts and keratinocytes into bovine collagen sponge scaffolds; however, these cells can only survive for less than 2 months *in vivo*, making it a temporary solution for wound repair (Stojic et al., 2019). To overcome this challenge, Amarantus Bioscience (San Francisco, CA) came up with an alternative approach to use autologous cells in a collagen sponge (Boyce et al., 2017). The majority of epidermal substitutes are made from cultural epithelial autografts (CEA), in which the patient’s cells are extracted, grown in culture, and then reapplied to the injured spot (Barret et al., 2000; Brockmann et al., 2018). Despite its extensive use, there is still ongoing development of CEA for improved physiological integration with the underlying dermis to reduce the production time of grafts *in vitro* (Dreifke et al., 2015).

Other than epidermal grafts, dermal substitutes received industrial attention in skin regeneration treatment. Although dermal replacements frequently necessitate two-step operations (followed by grafting of a CEA or other epidermal product), regulatory approval is easier for dermal replacements than for full-thickness skin substitutes that necessitate cell seeding, which makes regulation more severe (Savoji et al., 2018). Furthermore, many existing skin substitutes are costly due to high production costs (particularly those needing human cell culture), and those utilising allogeneic cells or ECM pose a risk of infection (Przekora, 2020). A total of sixteen well-designed clinical studies and ongoing clinical trials were identified in 2019. Table 2 summarizes the previous randomised clinical trials (RCTS) and ongoing clinical trials on skin substitutes. From sixteen RCTs, thirteen RCTs compared the efficacy of acellular skin substitute with standard of care such as sharp debridement, pressure redistribution support, glucose control, compression bandages, infection control, offloading, and daily dressing changes, while three studies investigated the safety and efficacy of cellular dermal substitutes. Furthermore, the lack of rigorous, well-controlled clinical trials of these products in this category may add to the paucity of clinical data for cellular dermal substitutes.

**TABLE 2:** Skin substitutes compared with standard of care in 16 RCTs.

Skin substitute	Category	Study Comparator(s)	Wound type	References
Affinity®	Cellular dermal	SOC	DFU	Serena et al. (2020)
Allopatch®	Acellular dermal	SOC	DFU	Zelen et al. (2018)
AmnioBand® Allograft Placental Matrix	Acellular dermal	SOC	DFU	DiDomenico et al. (2018)
AmnioExcel®	Acellular dermal	SOC	DFU	Snyder et al. (2016)
DermACELL®	Acellular dermal	SOC	VLU	Cazzell (2019)
Dermagraft®	Cellular dermal	SOC	VLU	Harding et al. (2013)
EpiCord®	Acellular dermal	SOC	DFU	Tettelbach et al. (2019a)
EpiFix®	Acellular dermal	SOC	DFU	Tettelbach et al. (2019b)
EpiFix®	Acellular dermal	SOC	VLU	Bianchi et al. (2018)
EpiFix	Acellular dermal	SOC	DFU	Zelen et al. (2013)
EpiFix	Acellular dermal	SOC	VLU	Serena et al. (2014)
Grafix®	Cellular dermal	SOC	DFU	Lavery et al. (2014)
Hyalomatrix® Wound Matrix	Acellular dermal	SOC	VLU	Alvarez et al. (2017a)
Integra® Dermal Regeneration Template	Acellular dermal	SOC	DFU	Driver et al. (2015)
MatriStem® Wound Matrix	Acellular dermal	SOC	DFU	Alvarez et al. (2017b)
Oasis® Wound Matrix	Acellular dermal	SOC	PU	Brown-Etris et al. (2019)

DFU, diabetic foot ulcer; PU, pressure ulcer; SOC, standard of care; VLU, venous leg ulcer.

None of the ESS shown in Tables 1, 2 appear to provide the optimal combination of biodegradable, biomimetic structure that promotes rapid wound healing and regeneration of native skin structure, including vascularisation, with little to no scarring. Furthermore, because they are made of fibroblasts and keratinocytes, the currently available skin substitutes lack the potential to form differentiated structures, such as hair and sweat glands; thus, it is critical to integrate additional cell types, such as endothelial cells, in ESS. As a result, the improvement of commercially accessible goods necessitates the development of multifunctional bioscaffolds with linked, porous, 3D geometry; mechanical strength; customised degradability; and biological signals for cells to repair and remodel tissue (Ghilardi et al., 2020).

## 5 Multifunctional bioscaffolds

A multifunctional bioscaffold is a porous, fibrous, or permeable 3D structure made of biomaterials infused with cells, bioactive molecules, secretomes, and antibacterial agents to facilitate the transport of body liquids and gases, promote cell interaction, sustain cell viability, and ECM deposition with minimal inflammation and toxicity while biodegrading at a controlled rate (Nikolova and Chavali, 2019). The 3D networks have unique properties that can imitate the skin ECM and have been shown to facilitate wound healing and skin regeneration (Zawani and Fauzi, 2021). These structures give mechanical stability and support to the tissue, as well as a vehicle for transferring bioactive compounds (drugs, antibiotics, growth factors, etc.) and templates for the attachment of genetically modified cells that generate new tissue regeneration centers (Chan and Leong, 2008; Hanczar et al., 2021). Moreover, the complex structure of the native tissue can be replicated using multifunctional bioscaffolds as potential tissue models, which makes biomaterials a component of prime importance for manufacturing multifunctional bioscaffolds. However, sometimes the multifunctional bioscaffolds present an associated risk of toxicity. Various scaffold materials showed different toxicity and pharmacology effects. Hence, the toxicity and biocompatibility tests are needed to evaluate scaffold material in mediating cell proliferation and differentiation, secreting ECM and carrying biomolecular signals for cell communication (Rahyussalim et al., 2017). Kamal et al. stated that a scaffold is considered toxic if it inhibits more than 50% of cell proliferation. The least inhibitory value means the scaffold is not toxic (Kamal et al., 2013).

### 5.1 Biomaterials used in developing multifunctional bioscaffolds

**FIGURE 3:**
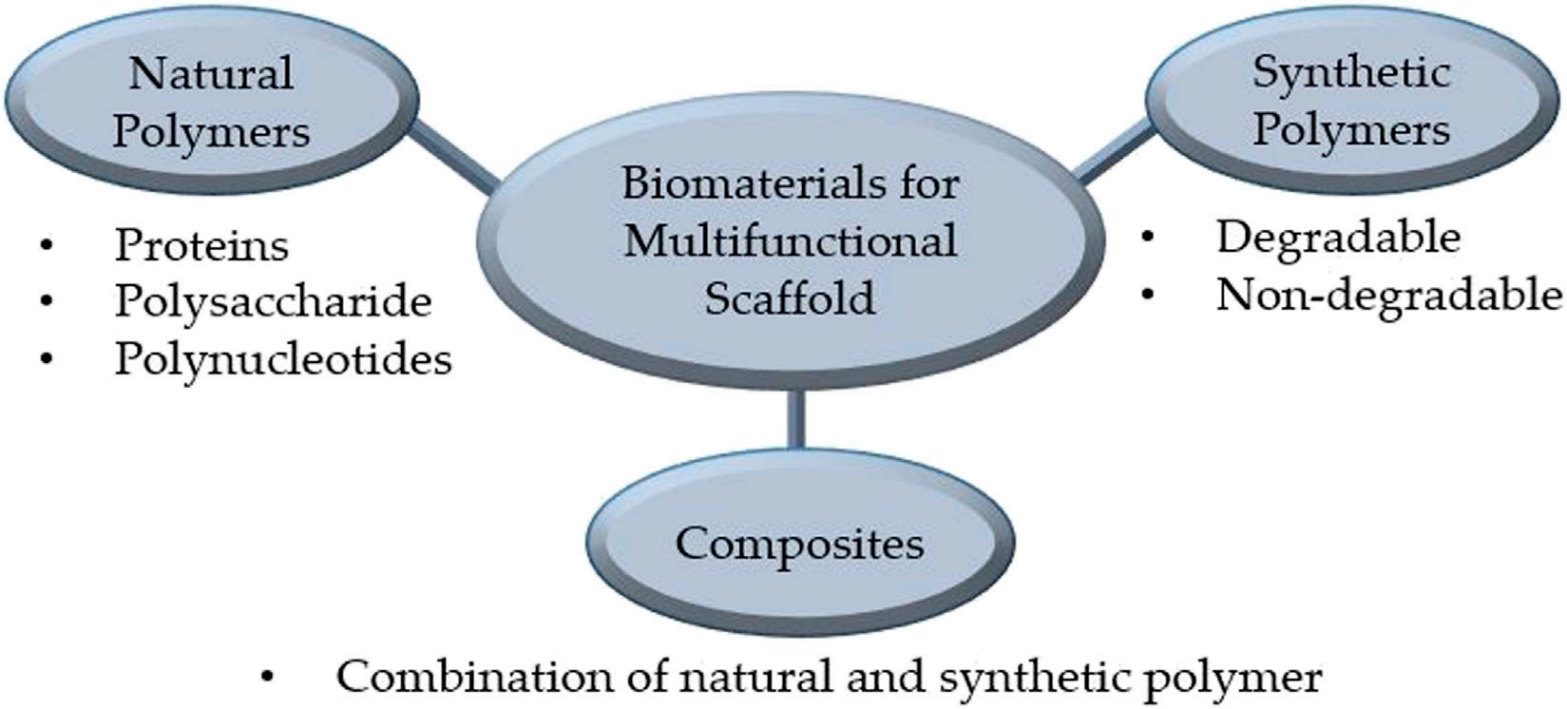
Type of biomaterials utilised for multifunctional scaffold manufacture.

Biomaterials used for manufacturing multifunctional bioscaffolds in skin tissue engineering can be classified into natural and synthetic polymers, and composites (Figure 3). Currently, the construction of multifunctional bioscaffolds focuses on biodegradable biomaterials that do not require extraction from the organism (Litowczenko et al., 2021). Table 3 outlines the major kinds of biomaterials employed in the manufacturing of scaffolds with multifunctional properties, as well as their fabrication process and common use. Each biomaterial has unique physical, chemical, and mechanical properties, necessitating specific production procedures to manage 3D shapes and geometry. The manufacturing procedures for multifunctional bioscaffolds are selected based on the scaffold’s specific requirements, the material of interest, and machine limitations (Mazzoni et al., 2021). The emergence of computer-aided design (CAD) software and rapid prototyping have paved the way for multi-bio development with macro, micro, and nano-architecture (Nikolova and Chavali, 2019). Moreover, using acquired patient data and advanced fabrication technology, the manufacture of multifunctional bioscaffolds could be personalised by fabricating a unique 3D model with specific geometry containing functional molecules compatible with multiple biomaterials and cells (Litowczenko et al., 2021; Moysidou et al., 2021). Table 4 outlined the fabrication techniques for multifunctional bioscaffolds with their advantages and disadvantages.

**TABLE 3:** Biomaterials are used in manufacturing multifunctional scaffold along with their application and fabrication technique.

Biomaterials for multifunctional scaffold			Application	Fabrication	References
Polymers	Natural	Proteins (actin, collagen, fibrinogen, gelatin, keratin, silk)	Regeneration of connective tissue	- Solvent casting	Sheikholeslam et al. (2018); Nikolova and Chavali (2019); Riha et al. (2021)
				- Inkjet printing	
		Polysaccharides (agarose, alginate, cellulose, chitin, chitosan, dextran, glycosaminoglycan, hyaluronic acid) Polynucleotides (DNA, RNA)	Decellularised living tissue/organ	-Particle aggregation	
			Drug delivery	- Micro moulding	
				- Photolithography	
				- Emulsification	
				- Electrospinning	
			Gene therapy	- Cryo-gelation	
				- Sol-gel	
	Synthetic	Degradable (polyesters, polylactones, polycarbonates, polyanhydrides, polyphosphazenes)	Drug delivery system	- Stereo-lithography (SLA) -Electron beam melting (EBM) -Selective laser sintering (SLS)	
			Implants		
		Non-degradable (polyacrylic acid, polyurethane, polymethylacrylate, polyether)	Implants	- Polyjet	
				- Electrospinning	
				- Phase separation	
				- Freeze drying	
				- Gas foaming	
Composites	Semi synthetic/Combinational polymers		Skin tissue repair and regeneration	- Freeze drying	Sheikholeslam et al. (2018); Nikolova and Chavali (2019); Riha et al. (2021)
				-Stereolithography (SLA)	
				-Fused deposition modelling (FDM)	

**TABLE 4:** Advantages and disadvantages of different fabrication methods for multifunctional bioscaffolds.

Fabrication methods	Advantages	Disadvantages	References
Electrospinning	- Possible to fabricate fibrous scaffold with fiber diameter ranging from microns to nanometers	- No shapes other than cylinder and sheets are possible	George et al. (2020); Park et al. (2021)
		- Limited of cells seeding	
Solvent casting	- Technically easy	- Only creates thin sheets of material	Lade Milind et al. (2013); Dong et al. (2020)
	- Better uniformity of thickness and better clarity than extrusion	- The polymer must be soluble in a volatile solvent or water	
3D-bioprinting	- Good mechanical properties	- Viscosity and temperature of materials	Bishop et al. (2017); Soori (2019)
	- Wide range of material choice	- Expensive equipment	
	- High resolution		
Freeze drying	- Pore structure with high interconnectivity	- Insufficient mechanical integrity	Thavornyutikarn et al. (2014); Nune et al. (2017)
	- Good porosity	- Small pore sizes	
	- Simple and cost-effective	- Uniform porosity cannot be maintained	
Gas foaming	- High level of pore interconnectivity	- Unsustainable processing	Udeni Gunathilake et al. (2017); Mirtaghavi et al. (2020)
		- Low kinetic stability	
Phase separation	- Desirable structural control	- Not user friendly	Altuntaş et al. (2017); Ganesan et al. (2018)
	-Easily combine with other fabrication technology	- Limited solvent choice	

Besides, the advent of modern techniques such as electrospinning and 3D printing have revolutionised the development of multifunctional bioscaffolds at a minimal cost, making it affordable for users (Masri et al., 2022). The minimal cost of manufacturing refers to printing 5,000 pieces of a physical library of mix-and-match channel scaffolds (100 μm) for USD$ 0.50 and making it available for researchers who lack access to suitable technology (Felton et al., 2021). Such proof was obtained in a study conducted by Felton (Felton et al., 2021) and his team, which revealed that it is possible to produce a 5000-piece library of microchannel modules using the 0.4 mm nozzle for less than USD$ 1.50. Recently, the significant demand for biomaterials has shown a gradual increase in the clinical industry to support the current standard treatment regarding cutaneous wound healing. Contributing to the high demand for therapeutic implants biomaterial usage has reached an annual growth rate of 16%, creating an estimated global market for biomaterials worth USD$ 207 billion by 2024 (Naomi et al., 2020). In addition, Smandri et al. reported the importance of many natural materials that could be introduced as bioinks for a better therapeutic approach in wound healing (Smandri et al., 2020). Still, the printable quality of the currently available bioinks demonstrated shortcomings in the physicochemical and mechanical properties of the printed bioscaffolds. It is necessary to obtain optimum pore sizes and porosity that will allow the migration of cells (Masri and Fauzi, 2021).

### 5.2 Multifunctional bioscaffolds in wound healing treatment

**FIGURE 4:**
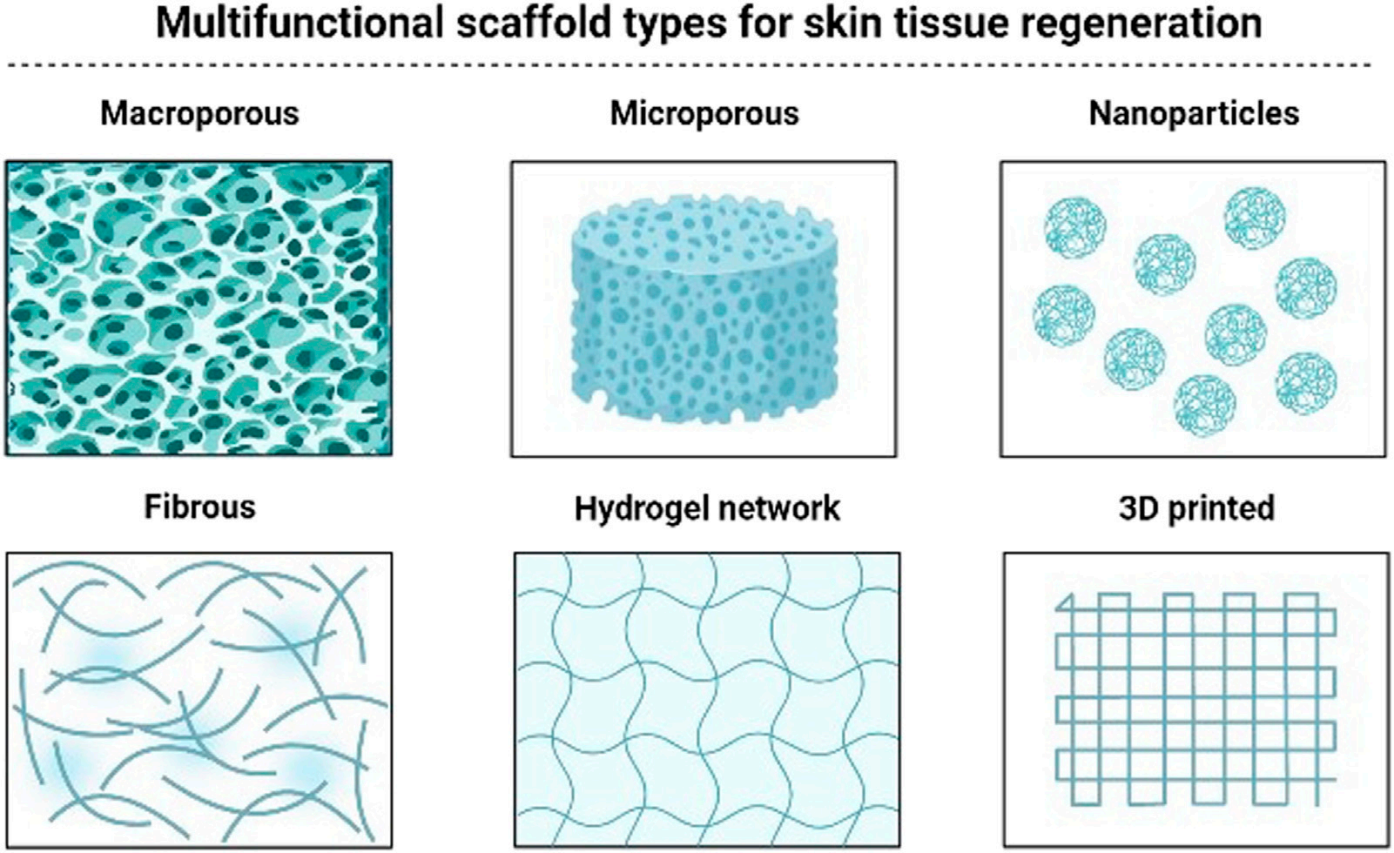
Types of multifunctional bioscaffold for wound healing purposes.

In recent years, increased research efforts have been made to construct multifunctional bioscaffolds comprising signaling components and active compounds for tissue engineering applications. Figure 4 presented several types of bioscaffold for the purpose of wound healing. These bioscaffolds’ ability to contribute to wound healing applications by encouraging tissue development and regeneration has been investigated primarily *in vitro* and *in vivo* research. Table 5 summarises the use of multifunctional bioscaffolds in wound healing applications along with their treatment outcomes. Yang et al. created a lidocaine hydrochloride (LID) and mupirocin loaded (Yang et al., 2020b), chitosan/polycaprolactone (CSLD-PCLM) nanofiber with variable dual drug release and multifunctional properties such as hydrophilicity, absorbing capacity, cytocompatibility, and antibacterial functions. So, it can improve wound healing conditions in both *in vitro* and *in vivo* models. The MTT assay in the *in vitro* study showed improved proliferation of human dermal fibroblasts after 2 days of culturing compared to the control group. However, after 3 days of culture, no significant difference was seen between the control group and the CSLD-PCLM scaffold. The study also showed 94.4% contraction of full-thickness excisional wound created in rats on day 7, followed by complete wound closure on day 14. Also, on day 14, similar *in vivo* wounds treated with solely CSLD and PCLM had 2% and 4.1% required closure, indicating the importance of the CSLD-PCLM nanofiber scaffold in facilitating quicker wound healing. Besides, the flexibility of electrospun materials contributed to 73% LID (the drug that reduces pain at the wound site in the early stages) release in the first 30 min and 28.7% mupirocin (antibacterial agent) within 24 h from the CSLD-PCLM scaffold.

**TABLE 5:** Multifunctional bioscaffold in wound healing application along with its treatment outcome.

Scaffold types	Fabrication approach	Study type	Application	Correction time	Multifunctional treatment outcome
Chitosan/polycaprolactone nanofibrous scaffold Yang et al. (2020b)	Electrospinning	*In vitro*	Human dermal fibroblast	3 days	Improved cell proliferation, and viability
		*In vivo*	Full-thickness wound in rat	14 days	Synergistic coagulation ability, antimicrobial activity, granulation tissue and collagen production, and re-epithelialisation were demonstrated
Chitosan/polycaprolactone nanofibrous scaffold Li et al. (2018c)	Electrospinning	*In vitro*	Human dermal fibroblast	2 days	Improved cell proliferation, and viability
Collagen/chitosan-glucan composite scaffold embedded with aloe-vera Abdel-Mohsen et al. (2020)	Lyophilization	*In vitro*	Normal human dermal fibroblast	4 days	Improved cell viability, and biocompatibility
		*In vivo*	Full-thickness wound in rat	8 days	Enhanced inflammation, proliferation and remodeling along with antibacterial property
Gallium mesoporous bioactive glass/chitosan composite scaffold Pourshahrestani et al. (2017)	Lyophilization	*In vivo*	Full-thickness wound in rat	8 days	Enhanced inflammation, proliferation and remodeling along with antibacterial property
Chitosan hydrogel inverse opal particles Chen et al. (2018)	Polymerization	*In vivo*	Acute excisional wound infection in rat	7 days	Enhanced angiogenesis, collagen deposition, granulation-tissue development, and inflammation reduction
Alginate/chitosan/fucoidan porous scaffold Hao et al. (2020)	Freeze drying	*In vitro*	Human gingival fibroblast	Not stated	Promoted adhesion and spreading of human gingival fibroblasts ensuring good biocompatibility and exhibited hemostatic and antibacterial abilities
		*In vivo*	Full-thickness wound in rats	9 days	Promoted re-epithelialisation, collagen formation in the dermis, and enhanced vascularisation and hair follicle regeneration
Type I collagen peptide and nitrous oxide releasing silk fibroin nanofibrous scaffold Ramadass et al. (2019)	Electrospinning	*In vitro*	NIH3T3 fibroblast	1, 3 and 5 days	Cells appeared to adhere well, had typical morphology on the surface, and were antibacterially efficient
Bioactive antibacterial hydrogel scaffold with exosome release Wang et al. (2019)	Schiff base reaction	*In vivo*	Diabetic full- thickness cutaneous wound in mouse	21 days	Angiogenesis was stimulated, and multifunctional qualities such as antibacterial activity, quick hemostatic ability, self-healing behavior, and UV shielding performance were demonstrated
Heparin-grafted aligned poly(lactide-co-glycolide)/curcumin nanofibrous scaffold Liao et al. (2021)	Electrospinning	*In vitro*	Human skin fibroblast cells (HS68)	1 day	Protection from induced oxidative stress and increased migratory ability
		*In vivo*	Diabetic wound in rats	14 days	Increased angiogenesis and collagen deposition, as well as accelerated re-epithelialisation

In addition to the mentioned works, one well-known biomaterial used for wound healing research is collagen, which can be classified into four main types: type-I, II, III, and IV. Fauzi et al. introduced collagen-type -I- based scaffolds for tissue engineering applications. The extraction of the collagen-type-I from the ovine tendon (OTC-1) demonstrated a high yield and comparable characteristics chemically with the commercial collagen-type-I (Amirrah et al., 2022; Salleh et al., 2022). In the previous study, the physicochemical properties of OTC-1 were evaluated with Fourier transform infrared spectrometry (FTIR), X-ray diffraction (XRD) and Energy dispersive X-ray spectroscopy (EDX) (Fauzi et al., 2016). Basically, the OTC-1 has the ability to be fabricated into 3D scaffolds and can be moulded into various types including sponge, film, and hydrogel that are biocompatible. Therefore, they are suitable to be used as scaffolds for developing tissue substitutes for *in vitro* tissue models, *in vivo*, and clinical applications. However, a study reported that the mechanical strength of the OTC-1 sponge crosslinked with genipin was significantly higher in terms of Young’s modulus (0.8290 ± 0.10 Gpa) and tensile strain (42.7% ± 1.59%) compared to the other crosslinked scaffolds (Busra et al., 2017). By *in vitro* studies, OTC-1 demonstrated no toxic effects on cells as it promoted higher cell attachment and proliferation towards both primary human epidermal keratinocytes and dermal fibroblasts. The efficacy of OTC-1 sponge crosslinked with genipin was evaluated in an *in vivo* full-thickness skin model. The results revealed no sign of immune response and the wound presented enhanced healing with superior skin maturity and microstructure features (Mh Busra et al., 2019).

Several studies have examined the effects of chitosan (CS) and polycaprolactone (PCL) on fibroblast cell proliferation (Shalumon et al., 2011; Mosallanezhad et al., 2022; Querido et al., 2022). Li et al. (Li et al., 2018b) investigated the properties of a double-layered fibrous PCL mat blended with CS, which demonstrated significant cell proliferation with spreading cellular morphology *in vitro*, whereas a PCL fibrous mat without CS showed minimal cell adherence. The double-layered multifunctional bioscaffolds were non-toxic to human dermal fibroblasts. Live/dead cell assay presented green fluorescence staining after indicating live cells without toxicity after 24 h of incubation. Moreover, MTT assay showed no inhibition of cell viability after 72 h of incubation which is attributed to the addition of CS.

Ozkan and Turkoglu Sasmazel (Ozkan and Turkoglu Sasmazel, 2016) demonstrated similar results, whereby the mouse fibroblast line (L929) showed improved cell adhesion and proliferation within a PCL/CS/PCL layer-by-layer hybrid scaffolds. The results aligned with Yang and his team (Yang et al., 2020b), where the bioscaffolds described the highest wound contraction area (94.4%) in rats on day 7 and was completely closed after 7 days. The improved efficiency of the scaffold in wound healing is due to the presence of CS and PCL. Positively charged CS acts as a hemostatic agent, promoting erythrocyte aggregation, improved platelet adhesion, full blood coagulation, homeostasis, and serving as a carrier for antibacterial drug dressings (Feng et al., 2016; Zhang et al., 2019a); whereas, PCL carried out the controlled release of mupirocin (antibacterial agent) preventing bacterial growth (Yang et al., 2020b). Furthermore, cell viability and density improved with time, and the bioscaffolds had no harmful effects on cells for 7 days, according to the absorbance value of the MTT cell viability assay (Podgórski et al., 2022). Besides, the nanofibrous nature of the scaffold constructed via electrospinning allowed drug loading and encapsulation of mupirocin, followed by its controlled release (Saghazadeh et al., 2018). Another study by Abdel-Mohsen and his co-workers (Abdel-Mohsen et al., 2020) stated that a novel multi-functional collagen (CO)/chitosan-glucan (CSGC) hollow fiber/(CO)/aloe-vera (AV) composite bioscaffolds demonstrated increased swelling, hydrolytic degradation, controlled porosity, and hemostatic properties. Within 8 days of administration to the full-thickness wound region of rats, there was increased fibroblasts migration during the creation of granulation tissue, collagen, and re-epithelialisation in proliferative phases. Furthermore, the composite multifunctional scaffold also maintains optimum hydration of the exposed tissue, reducing wound healing time. Besides, the scaffold further demonstrated improved viability and biocompatibility of normal human dermal fibroblast *in vitro*. The results are in coherence with Chowdhury and his team (Chowdhury et al., 2011) whereby a porous sheep collagen sponge successfully fabricated using freeze-drying method showed no cytotoxic and genotoxic effect towards human dermal fibroblasts, thus ensuring biocompatibility.

In addition, *in vitro* study of gallium-incorporated mesoporous bioactive glass/chitosan composite multifunctional bioscaffolds constructed via lyophilisation by Pourshahrestani and her team (Pourshahrestani et al., 2017), exhibited increased thrombus generation, blood clotting, and platelet adhesion and aggregation after 4 days of culture. Moreover, the highly ordered mesoporous channel structure of the composite bioscaffold provides high porosity, huge surface area, and pore volume, which allows easy incorporation and delivery of low concentration (1 mol%) of therapeutic gallium ion, improving the biodegradability and biocompatibility of the bioscaffold. Besides, *in vitro* cytotoxicity evaluation of the scaffold demonstrated non-cytotoxicity towards human dermal fibroblasts and exhibited a large number of viable cells on the surface of the bioscaffold after 4 days of culture, attributing its cytocompatibility towards human dermal fibroblasts (Wang et al., 2013).

Apart from fibrous and composite multifunctional bioscaffolds, Chen and his fellow members (Chen et al., 2018) reported that chitosan hydrogel inverse opal particles loaded with fibroblast growth factor demonstrated facilitated cell distribution and migration, followed by improved angiogenesis, collagen deposition, granulation-tissue formation, and reduced inflammation within 7 days in acute infectious rat wound. The study also suggests that the multifunctional bioscaffold supports the effective transfer of oxygen, nutrients, and metabolic wastes following the release of growth factors, which is crucial for maintaining high viability of the tissue during the wound healing process prior to angiogenesis. Moreover, another study by Hao and his team (Hao et al., 2020) used a multifunctional composite sponge made of alginate/chitosan/fucoidan (ACF) developed through electrostatic interaction, Ca^2+^ crosslinking, and freeze-drying process, which showed improved wound healing properties both *in vitro* and *in vivo.* The study reported that the bioscaffold possessed flexible mechanical properties as its pore size and porosity could be tailored using various fucoidan and alginate/chitosan concentrations. The study found that 10% fucoidan had better hemostatic and antibacterial activities *in vitro* than 30% fucoidan. Furthermore, in a full-thickness rat wound model, ACF bioscaffold with 10% fucoidan significantly promoted dermal re-epithelialisation and collagen formation, enhanced vascularisation by upregulating the specific protein expression of CD31, and hair follicle regeneration, as well as suppressed inflammation by downregulating TNF-specific protein expression.

Apart from the excisional wound model, bioscaffolds with multifunctional properties also show promising results in diabetic wound healing. Ramadass and his co-workers (Ramadass et al., 2019) developed a type I collagen peptide (CP) and S-Nitrosoglutathione (GSNO) incorporated with silk fibroin-based multifunctional scaffold to improve diabetic wound healing in clinical and experimental studies. The study demonstrated that NIH3T3 cells continued to proliferate on the surface of the scaffold, implying that the scaffold was non-toxic to fibroblast cells. In this scenario, the use of type I collagen in smaller peptide form may facilitate in the regeneration of lost ECM by providing better bioavailability to the wound bed compared to parent collagen as well as benefit the cell-matrix interaction via improved hydrophilicity, whereas, S-Nitrosoglutathione formulations could enhance the microvascular blood supply in both clinical and experimental studies (Ramadass et al., 2019). Moreover, the nanofibrous structure made of silk fibroin with regenerative and functional components serves as a potential delivery vehicle for transporting functional moieties to the wound site in treating non-healing diabetic ulcers by providing mechanical support, biocompatibility, and a moist healing environment (Chouhan et al., 2017). Similarly, Wang et al. with his team (Wang et al., 2020) fabricated injectable hydrogel-based multifunctional bioscaffolds with self-healing, antibacterial, hemostatic, and UV shielding properties which can facilitate the release of therapeutic exosome promoting diabetic wound healing. The bioscaffold aided in cell proliferation, migration, and angiogenesis in newly formed tissue, resulting in the production of granulation tissue, collagen deposition, and remodeling, expediting diabetic wound healing and even restoring skin appendages in healed wounds. Exosomes (produced from mesenchymal stem cells) were used in this investigation because they include mRNA, miRNA, growth factors, and protein molecules that can be transported to target cells to facilitate intercellular communication and the physiologic wound healing process (Fadilah et al., 2022). Besides, wound healing was attributed to the bioscaffold’s fabrication and multifunctional properties, as the injected hydrogel nature of the bioscaffolds can absorb wound secretion, maintain a moist environment to keep a relatively favorable local environment for healing, and result in better healing compared to control (Hu et al., 2023; Liu et al., 2023; Wang et al., 2023). Furthermore, the multi-functional bioscaffold’s antibacterial and UV-shielding properties can protect the wound bed from bacterial invasion and light-induced damage, as demonstrated in earlier research (Wang et al., 2019; Li et al., 2022). Also, the bioscaffolds showed good biocompatibility *in vivo* as no hemorrhage and inflammatory infiltration was observed in the mouse model’s heart, lung, liver, and kidney (Li et al., 2023).

Nanofiber materials have often been reported as transporters for clinical drugs but face the limitation of burst releasing the drugs (Fadilah et al., 2019). The 3D scaffolds such as nanofibers might offer promising results to facilitate the restoration of target tissues. Moreover, the advances in nanotechnology have offered groundbreaking progress in the field of tissue engineering by providing a microenvironment for the induction of cell expansion and differentiation (Ahmadian et al., 2023). In another study, Liao et al. (2021) created an aligned poly(lactide-co-glycolic acid) (PLGA)/curcumin nanofibrous multi-bio with a high-density heparin surface coating to attract endogenous growth factor and facilitate curcumin release, which could serve as an exogenous factor for wound healing. According to the study, the multifunctional scaffold displayed good tensile strength, minimal cytotoxicity, and a sufficient water vapor transmission rate for use as wound dressings. Furthermore, the bioscaffold’s aligned nanofiber orientation may facilitate migration of fibroblasts and keratinocytes from the peripheral wound area to the center, as well as promote re-epithelization and collagen deposition, hence shortening wound healing time (Ottosson et al., 2017). Despite this, the multifunctional bioscaffolds demonstrated a faster rate of curcumin release than the control due to increased hydrophilicity, which contributes to a higher cell migration rate and induced oxidative stress protection of HS68 fibroblast cells *in vitro*. Furthermore, an *in vivo* investigation revealed a rapid rate of wound closure, quicker re-epithelisation, increased angiogenesis, and increased collagen deposition at the wound site (Liao et al., 2021). Furthermore, the rapid skin regeneration may be related to the multifunctionality of nanofibers, where grafted heparin attracted and stabilized the growth factors required for wound healing *in situ* (Veith et al., 2019), as well as relieving the high oxidative stress and inflammatory cascade caused by released curcumin during diabetic wound healing (Ranjbar-Mohammadi et al., 2016; Mohanty and Sahoo, 2017).

Recently, the combining NPs into bioscaffolds represents the targeted approach of advance treatment that has gain significant attraction to the existing therapeutics (Kalantari et al., 2020; Pormohammad et al., 2021). In addition to the bioscaffolds indicated in Table 5, inserting metal-based NPs in scaffolds has sparked considerable interest in TERM. Depending on the application, many synthetic methods have been used to prepare NPs with low toxicity, contrasting agent properties, tailorable characteristics, targeted stimuli/response delivery potential, and precise control over behavior (via external stimuli such as magnetic field), which has found applications in various areas of TERM (Bahal et al., 2016; Mili et al., 2018). In animal models, for example, integration of chitosan (Kaparekar et al., 2020), gold (Nanda et al., 2022), and silver (You et al., 2017) based NPs in multifunctional bioscaffolds successfully increased re-epithelialisation, accelerated fibroblast cell migration, wound healing, and considerable wound contraction. Besides that, Sharma and Kaushal reviewed the formation of nanoparticles in green synthesised where they have played a significant role due to their higher reactivity and stability from different sources such as bacteria, fungus, algae, and plants (Sharma and NavneetKaushal, 2022). The nanoparticles can stimulate numerous cellular and molecular processes that aid in wound microenvironment via different mechanisms such as anti-inflammatory, antimicrobial, and angiogenic effects, possibly changing the milieu from non-healing to healing (Kushwaha et al., 2022). However, due to the prominent nature of the associated toxicity and environmental concerns contained in most of these conventional processes, the demand for more clean, trustworthy, eco-friendly, and biologically compatible procedures has limited their continuous usage.

Metal NPs can now be synthesised utilizing plant-mediated methods, which avoid many of the drawbacks of traditional synthetic methods. Bioresources are used as a scaffold to reduce and stabilise the materials, making them biocompatible with living cells (Jo et al., 2022). Many studies have been conducted in biochemical synthesis and analysis due to plants’ intrinsic ability to reorganise inorganic metal ions into NPs via organic processes. Several published research findings strongly suggest that natural-based products containing NPs are beneficial and safe for developing of scaffolds (Zhang et al., 2019b; Kaparekar et al., 2020; Konop et al., 2020; Fahimirad et al., 2021). The presence of beneficial phytochemicals inside the plants has been linked to these healing properties. However, there are still significant health concerns, particularly regarding toxicity due to the NPs’ uncontrollable use and release. As a result, the inclusion of NPs with natural-based biomaterials for scaffold construction should be addressed to make the use of slow-release NPs easier, safer, and more ecologically friendly to improve wound healing.

## 6 Challenges and improvement of multifunctional bioscaffolds towards future translational applications

Although a wide range of successes in wound healing and skin regeneration has been shown *in vitro* and *in vivo* in animal models using multifunctional bioscaffolds, almost no progress has been made in translating to human clinical trials towards commercialisation. This is due to the heterogenous nature of wounds among the patients depending on the considered pathology, which cannot be treated using a single dressing or scaffold that can meet the diverse needs of all wounds (Memic et al., 2019). Hence, treatment for skin regeneration should progress towards a personalised therapy-based multifunctional bioscaffolds production for tissue regeneration. Despite making considerable progress in multifunctional bioscaffolds research, multiple challenges must be resolved to make it available for clinical use. The significant hurdles include i) modulating the properties (structural and biomechanical), and degradation rate of multifunctional bioscaffolds depending on the intended use by optimising surface characteristics to improve cell adhesion and ECM deposition; ii) efforts in manufacturing multifunctional bioscaffolds for intended application, using various fabrication techniques by combining different biomaterials incorporated with bioactive molecules; iii) customising therapeutic approach via selection of biomolecules and optimising appropriate dose considering the level of skin damage and patient condition iv) increased vascularisation preventing localised necrosis, and implant failure minimising immune response; v) improving the clarity and accuracy of technology for producing a multifunctional bioscaffolds, and precisely replicating the composition of the ECM; vi) reducing the post implantation effects like immune rejection, and secondary damages; vii) minimising complexity in fabrication process; and (viii) industrial scale production and commercialisation outside the laboratory environment (Nikolova and Chavali, 2019). Hence, eliminating these challenges requires optimising the multi-bio architecture and providing a conducive biological environment for its effective outcome in skin regeneration treatment. In this scenario, using advanced technologies such as computational modelling, electrospinning, and 3D-printing for skin regeneration would optimise bioscaffolds depending on wound requirements.

Computational models can play an important role in designing multifunctional bioscaffolds since they can be used to determine the best mix of physical, chemical, and biological properties and give a precise simulation of wound healing and deformation (Chang et al., 2017). Prevailing computer-aided design (CAD) software is mainly used for manufacturing existing scaffolds, which emphasises geometric shapes; however, the CAD system cannot guarantee multifunctional capability as it is difficult to incorporate and optimise these attributes manually design (Leung et al., 2019). Researchers currently use CAD software coupled with finite element analysis (FEA) and a trail error approach to design multifunctional properties (Chang et al., 2017). Electrospinning has also received attention in the development of multifunctional bioscaffolds for wound healing applications and is already in use by companies to manufacture nanofiber-based wound dressings and skin substitutes for commercial purposes. This technology is used to fabricate biomimetic multifunctional bioscaffolds with growth factors, antimicrobial agents, anti-inflammatory drugs, and anesthetics which can be used for epidermal grafts (Memic et al., 2019). Recent studies by Li (Li et al., 2018c); Liao (Liao et al., 2021), Ramadass (Ramadass et al., 2019) and their co-workers have demonstrated satisfactory outcome in skin tissue regeneration when combined with stem cells, bioactive compounds, and nanomaterial. Nonetheless, the bulky and expensive setup of electrospinning devices limited its use in tissue engineering application. However, the recent advances in nanotechnology has allowed the manufacturing of light weight, battery operated and portable devices, which hold the potential of increased production and utilisation of electrospun nanofibers for clinical application (Memic et al., 2019).

Apart from electrospinning, the 3D bioprinting method has emerged as potential alternative technology which is used in fabricating more complex skin substitutes with precision by incorporating several cell types, bioactive molecules, and NPs in an automated way reducing cost and time in the manufacturing process (Jamróz et al., 2018). This new technology operates by precisely depositing biological agents in layers to form complex structures. The biological agents include using natural and synthetic biomaterials to form structural matrix, different types of cells, growth factors, antimicrobial or anti-inflammatory drugs, secretomes, and other bioactive materials (Leung et al., 2019). A patient with full-thickness burns or other deep skin damage may benefit from the implantation of a multifunctional scaffold made using 3D bioprinting because it allows for the precise placement of cells to repair damaged skin while minimising the number of surgeries and length of the patient’s stay. Additionally, it may be possible to manage the geographic integration of multifunctional scaffold and cells in 3D printed skin structures, which could result in a more effective system that speeds up regeneration while possibly requiring less intervention (Masri et al., 2022). Despite being a developed technology, 3D bioprinting suffers from multiple challenges which need to be resolved. Some of the hurdles include i) the capacity of 3D bioprinting to produce tissues at the human scale; ii) the reproducibility of the intricate structure resembling ECM; iii) the embedding of desirable cells capable of developing into a mature tissue; iv) the time spent to print out a clinically relevant skin construct; and v) the ethical, social, and regulatory issues associated with 3D printed skin (Kačarević et al., 2018). The urge to produce quick and instant bioengineered skin construct for immediate implantation gave birth to the idea of *in situ* bioprinting. In most cases, the application of 3D bioprinting under laboratory setting is carried out by printing the 3D skin under *in vitro* conditions, followed by post-processing and subsequent engrafting in animal models. However, in clinical setting this approach could give rise to potential complications like construct damage upon transportation or manipulation, and haphazard placement of a construct with a complicated 3D topology on the wound bed (Murphy and Atala, 2014; Singh and Jonnalagadda, 2020). Such complications can be avoided via *in situ* bioprinting whereby a printing device can directly graft 3D bio-printed skin on the patients’ wound site, regardless of the size using their body as a bioreactor to develop engrafted skin construct functionally (Manita et al., 2021).

Biological research may significantly influence the best multifunctional bioscaffolds design for skin regeneration to understand better the wound milieu (physical and biochemical) (Muzzio et al., 2021; Qin et al., 2022). Additionally, it is crucial to take caution when modifying the physicochemical characteristics of multifunctional bioscaffolds because their size, shape, composition, charge, and topography can affect the host’s inherent immunogenicity (Echeverria Molina et al., 2021). A chain of events known as the foreign body response (FBR) is triggered by the implantation of bioscaffolds *in vivo* and typically lasts for 1–2 weeks (Chen et al., 2019; Veiseh and Vegas, 2019; Capuani et al., 2022). For instance, the bioscaffold’s pore size can affect the development of fibrotic capsules and change the phenotype of macrophages, both of which are crucial to the healing of wounds. Increased pore size of electrospun bioscaffolds has been linked in studies to a change in the M2 macrophage phenotype (Garg et al., 2013; Sussman et al., 2014). Porosity may harm the mechanical strength of the construct, which may be inadequate for reproducing the strength of the native tissue, even though it can be used to foster a regenerative environment by modifying the macrophage phenotype. Additionally, via affecting FBR and fibrosis, scaffold size and shape have an impact on the phenotypes of responding immune cells. The host’s ability to recognise the implanted bioscaffolds and the propagation of foreign body reactions can both be impacted by changing its geometry. For example, it has been established that spherically shaped implants are necessary for resisting host fibrosis and that increasing implant size is insufficient to prevent FBR (Veiseh et al., 2015). Moreover, the chemical properties of multifunctional bioscaffolds can also have a significant impact on the host response. Carbodiimide or glutaraldehyde are crosslinking agents that strengthen ECM-based scaffolds and have been shown to encourage an early pro-inflammatory immune cell phenotype and inhibit macrophage ECM degradation (Brown et al., 2012; Sadtler et al., 2019). On the other hand, genipin is preferable to other plant-based crosslinkers due to its biological activities, such as antioxidant and anti-inflammatory which are key players in boosting skin wound healing (Utami Nike et al., 2022). The integration of natural-based antioxidant compounds in the implanted biomaterial has revealed an advancement in supporting and expediting tissue regeneration where the scaffolds should be able to maintain their antioxidant activity while facilitating skin tissue recovery. Fadilah et al. reviewed the antioxidant-based therapies by summarised the recent literature that reported the role of natural antioxidant-incorporated biomaterials in promoting skin wound healing and tissue regeneration (Fadilah et al., 2023). For future directions on antioxidant therapy, earlier intervention seems to have a higher chance of success. Antioxidants do not appear to be very effective when the disease is already well-established and compelling evidence shows that they are more effective in prevention rather than treatment. This leads to the assumption that the combination of different antioxidants compounds or materials might have a synergistic effect towards the treatment (Firuzi et al., 2011). Therefore, tissue repair and regeneration must build multifunctional bioscaffolds that actively modify the immune response rather than avoid or inhibit it. As such, researchers are working on developing new strategies at harnessing the immunomodulatory response with the help of advanced fabrication techniques via cautious morphology and architectural selection and design to promote multifunctional scaffold tolerance.

## 7 Conclusion and future perspective

In summary, the successful recovery of the complex wound healing process demands an orderly cascade of biological events that involves interaction of cells with their physical and biochemical environment to promote skin regeneration. It is a significant problem for clinicians to treat varied wound types; and therefore, an extensive understanding of wound types and appropriate skin grafts is required. Thus, this review has depicted an overview of the development of multifunctional bioscaffolds, which led to significant improvements in skin tissue engineering. Implantation of such scaffolds with multifunctional properties to the wounded area not only provide accurate moisture level and prevent bacterial infection but also contributes to the healing process, cell proliferation and ECM modelling due to the combined work of the polymeric constituents, architecture, and biological agents, as shown in several *in vitro* and *in vivo* studies summarised here. The review further emphasises that multifunctional bioscaffolds must not only be passive supports for stem cell activity after implantation to improve regeneration, but they can also be engineered in various ways to modify inflammatory response and influence stem cell activity. Though the wound healing efficacy of multifunctional scaffold has been tested primarily on excisional wounds created in animal models, its translational use for clinical application faces some challenges in terms of affordable cost, industrial-scale production, optimised fabrication technique, and off-the-shelf availability to meet high demands in hospitals. Hence, we suggest further investigation into advanced fabrication techniques such as computational designing, electrospinning, and 3D bioprinting to fabricate multifunctional scaffolds with long-term safety. Understanding immune cell scaffold cross-interactions and their implications for host response are equally critical. Finally, comparative studies on commercially available engineered skin substitute and multifunctional bioscaffolds used in various wound healing treatments and their long-term follow-up studies will help consolidate healing efficacy.
