# Supplementary figures and images for: Functionalised-biomatrix for wound healing and cutaneous regeneration: future impactful medical products in clinical translation and precision medicine
Source: Front Bioeng Biotechnol. 2023 May 24;11:1160577. doi: 10.3389/fbioe.2023.1160577 (PMC10245056; doi:10.3389/fbioe.2023.1160577)

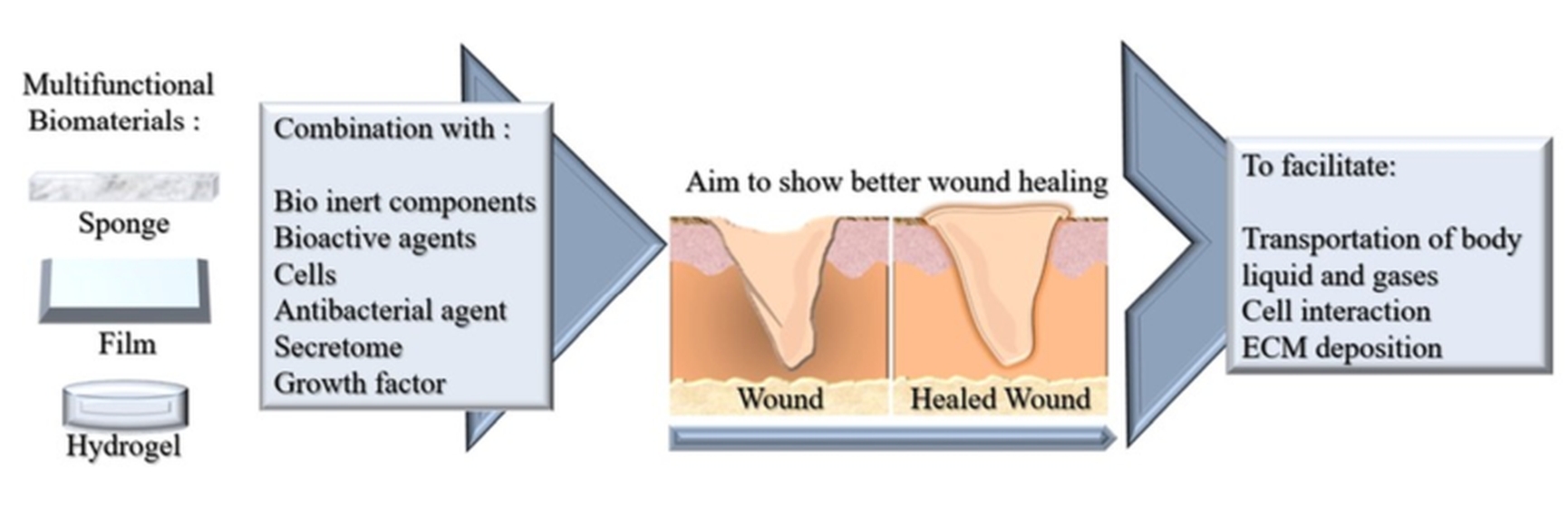

Supplement: Supplementary file 1 [file Image1.JPEG]
